# Growth mechanism and magnon excitation in NiO nanowalls

**DOI:** 10.1186/1556-276X-6-485

**Published:** 2011-08-08

**Authors:** Ashish Chhaganla Gandhi, Chih-Yeh Huang, Chun Chuen Yang, Ting Shan Chan, Chia-Liang Cheng, Yuan-Ron Ma, Sheng Yun Wu

**Affiliations:** 1 Department of Physics, National Dong Hwa University, Hualien 97401, Taiwan; 2 Department of Physics, Chung Yuan Christian University, Chungli 32023, Taiwan; 3 National Synchrotron Radiation Research Center, Hsinchu 30076, Taiwan

**Keywords:** magnetic oxides, nanocrystalline materials, confocal Raman scattering, short-circuit diffusion, magnon excitation

## Abstract

The nanosized effects of short-range multimagnon excitation behavior and short-circuit diffusion in NiO nanowalls synthesized using the Ni grid thermal treatment method were observed. The energy dispersive spectroscopy mapping technique was used to characterize the growth mechanism, and confocal Raman scattering was used to probe the antiferromagnetic exchange energy *J*_2 _between next-nearest-neighboring Ni ions in NiO nanowalls at various growth temperatures below the Neel temperature. This study shows that short spin correlation leads to an exponential dependence of the growth temperatures and the existence of nickel vacancies during the magnon excitation. Four-magnon configurations were determined from the scattering factor, revealing a lowest state and monotonic change with the growth temperature.

PACS: 75.47.Lx; 61.82.Rx; 75.50.Tt; 74.25.nd; 72.10.Di

## Introduction

Nanomaterials aroused considerable interest in the twentieth century because it was found that nanoparticles (0D, 1D, and 2D) ranging in size from 1 to 100 nm exhibit novel properties which are significantly different from properties of the bulk material. These properties arise from different nanoscale effects such as the quantum size effect, surface effect, finite size effect, and macroscopic quantum tunnel effect [1-4]. Most of the anomalous behaviors are observed in the nano-region of antiferromagnetic- and ferromagnetic-amorphous and crystalline nanoparticles [4,5]. For example, bulk NiO has a two-sublattice antiferromagnetic crystalline structure, but on the nanoscale, it has a many-sublattice antiferromagnetic crystalline structure [6] along with novel mechanical, optical, electronic, magnetic, and thermal properties. It has potential applications for catalysts, battery electrodes, gas sensors, electrochemical films, and photoelectronic devices [7-11]. There have been numerous qualitative and quantitative theoretical and experimental investigations of NiO materials using Raman and neutron scattering techniques [12-16]. Raman spectroscopy has been utilized to study magnon and phonon excitation in bulk NiO and NiO nanoparticles below the Neel temperature [2-4]. The dominant superexchange interactions [17] between the next-nearest-neighboring (NNN) Ni ions in the linear atomic chain Ni^2+^-O^2-^-Ni^2+ ^in a multisublattice magnet have been investigated. Analysis of the short range multimagnon interaction and even determination of the configuration of the magnetic structure is becoming possible. Vertical and interconnected two-dimensional nanostructures, such as NiO nanowalls have been produced with various fabrication methods [18,19]. Such structures have recently aroused a great deal of interest due to their application in high performance Lithium ion batteries [20]. Two-dimensional NiO nanowalls are considered to be ideal for the study of short-range magnetic ordering due to their high surface to volume ratio, interconnecting behavior and open-edge geometry [19,21].

In this study, we report on the successful synthesis of well-aligned and interconnected 2D nanowalls of NiO by a simple thermal treatment method for oxidizing the Ni grid. The energy dispersive spectroscopy (EDS) mapping technique is used to characterize the formation of NiO nanowalls at various annealing temperatures. This process can also be well described using a short-circuit diffusion simulation. The interaction of light with the spin degrees of freedom gives two-magnon (2 M) and four-magnon (4 M) shifts in the Raman energy spectra. The energy shift in the two-magnon peaks is due to the lowering of local symmetry at the Ni^2+ ^sites caused by chemical substitution and vacancies. The magnon configurations can be determined because of the dependence of the two- and four-magnon peaks on the growth temperatures.

## Experimental details

**Figure 1:**
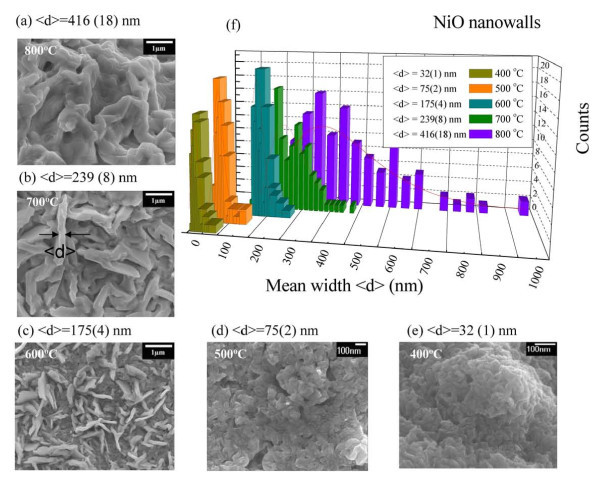
**SEM images of the NiO nanowalls**. **(a-e) **SEM images of the NiO nanowalls with various mean widths <*d *> which were synthesized at various annealing temperatures. **(f) **Distributions of mean nanowall width obtained from a portion of the SEM image of NiO nanowalls. The solid lines represent the fitting curves assuming the log-normal function.

A series of template-free NiO nanowalls with various grain sizes were fabricated. The samples were prepared by a process where a pure Ni grid (200 mesh) was placed in a ceramic boat inside a quartz tube, which was then evacuated to about 10^-3 ^Torr using a mechanical pump. The samples were then heated in a tube furnace at about 200°C for 2 h for degassing before being heated to various temperatures ranging from 400°C to 800°C for 3 h in a mixed argon (100 sccm) and oxygen (10 sccm) gas. The morphology and structures of the prepared samples were characterized using field-emission scanning electron microscopy (JEOL JSM-6500F, JEOL Ltd., Japan). For the SEM analysis, some of the nanowalls were transferred by gently sliding them onto a commercially available cooper grid with a carbon film. The SEM images in Figure 1a, b, c, d, e shows the surface morphology of NiO nanowalls with various sizes synthesized at *T*_A _= 800°C, 700°C, 600°C, 500°C, and 400°C, respectively. It can be seen that the NiO nanowalls form homogeneously on the nickel grid substrate. The mean sizes <*d *> of the nanowalls were calculated and defined from their width and grain, as shown in Figure 1a, b, c, d, e, respectively. The distribution of the mean width <*d *> of the NiO nanowalls as seen in Figure 1f is quite asymmetric, assuming a log-normal function distribution. The solid curves represent the fitting curve assuming log-normal distribution function. The log-normal distribution is defined as follows:  where <*d *> is the mean value and *σ *is the standard deviation of the function. The mean NiO nanowall widths <*d *>, as determined from the SEM images and described by the fit of the log-normal function, were approximately 32(1), 75(2), 175(4), 239(8), and 416(18) nm, respectively. The corresponding fitting parameters are presented in Table 1. The value of the standard deviation of fitted function which is less than 0.5 for all annealing temperatures indicates that the distribution is confined to a limited range. There is a surprising contrast in sample color. The resultant NiO nanowalls can be either "black" (*T*_A _= 400°C and 500°C) or "green" (*T*_A _= 600°C-800°C), containing nickel vacancies, depending on the growth temperature and oxygen supplementation given various concentrations of nickel vacancies.

**Table 1:** Fitting parameters

*T*_A _(°C)	Width <*d *> (nm)	*σ *(nm)	Area
400	32 (1)	0.32 (3)	329 (24)
500	75 (2)	0.21 (2)	591 (54)
600	175 (4)	0.32 (7)	946 (102)
700	239 (8)	0.34 (3)	2,327 (196)
800	416 (18)	0.45 (6)	5,593 (432)

Summary of the log-normal distribution function fitting parameters obtained from FE-SEM images of the NiO nanowalls.

## Growth mechanism of NiO nanowalls

### Analysis of morphology by FE-SEM

**Figure 2:**
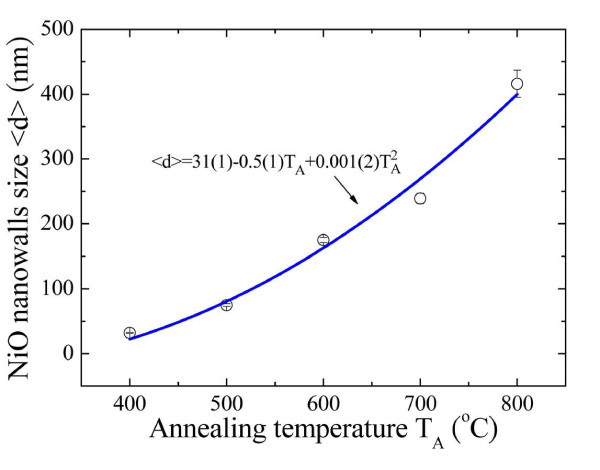
**Relation between the annealing temperature *T*_A_ and the mean nanowall width <*d *>**. The growth temperature *T*_A _dependence of the mean nanowall width <*d *>, where the solid curve shows the fit to the parabolic law and the fitted values.

The SEM results showed a significant increase in the average NiO nanowall width <*d *> from 32(1) to 416(18) nm, with a clear dependence on temperature. It is still unclear, however, under which condition grain growth occurs, and whether it is mainly caused by thermal activation or induced by the annealing time. From the macroscopic view, in this present study, the growth temperature of NiO nanowalls was confined to between 400°C and 800°C, which is 0.275 and 0.55 times the melting point of Ni melting (1,453°C), following the parabolic rate law of Wagner's scaling theory [22,23]. Figure 2 shows relation between the annealing temperature *T*_A _and the mean nanowall width <*d *>, where the solid curve indicates the fit to the parabolic law and the fitted values obtained. The phenomenon of NiO nanowall growth following recrystallization at the grain boundaries has been well documented in the previously report [24]. For example, Upadhyay and colleagues [25] studied the effect of sintering temperature on grain boundary character distribution in pure nickel. They report on observations that the grain boundary distribution and grain growth are correlated well with the accompanying microstructural changes. The annealing of the Ni grid led to the oxidation of the surface Ni, resulting in the formation of a polycrystalline nanosized layer of NiO grains at the surface. Diffusion of oxygen into the Ni voids takes place through these microcracks, resulting in the further formation of NiO nanowalls at the boundaries of the grains. A further increase in annealing temperature results in the formation of a nanowall-type structure over the Ni surface. The width of the nanowalls increases with increasing annealing temperature and at a sufficiently high temperature will merge together. The annealing of Ni grid results in the formation of NiO grains at a lower *T*_A _and a merged nanowall film at higher growth temperature, respectively.

### Annealing temperature dependence of X-ray diffraction

**Figure 3:**
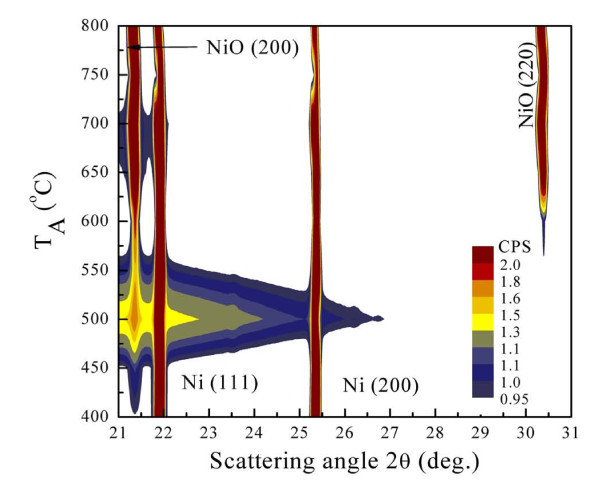
**Two-dimensional map of intensity and *T*_A _dependence in X-ray diffraction patterns taken at room temperature**.

**Figure 4:**
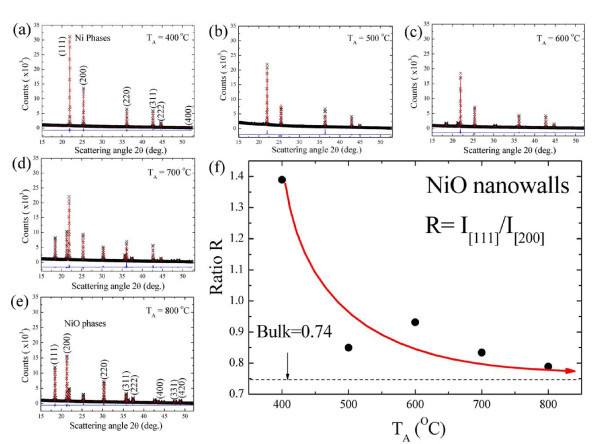
**The X-ray diffraction patterns of the NiO nanowalls**. **(a-e) **X-ray diffraction and Rietveld refinement at various *T*_A_; **(f) **ratio of the integrated intensity of [1 1 1] and [2 0 0] of NiO nanowalls as a function of *T*_A_. The ratio of the integrated intensity [1 1 1] and [2 0 0] is noticeably higher than the standard value of 0.74 in bulk NiO bulk (dashed line) at lower *T*_A _and closer to the standard value at *T*_A _= 800°C.

The high energy synchrotron radiation X-ray diffraction (SR-XRD) technique must be employed for detailed investigation of the microcrack-induced strain leading to the formation of NiO nanowalls because small changes in strain are undetectable by the usual XRD techniques. An analysis of the crystalline properties was carried out by XRD at the National Synchrotron Radiation Research Center in Hsinchu, Taiwan (*λ *= 0.7749 Å) using SR-XRD with a BL01C2 beam line. Figure 3 shows the *T*_A _dependency of the X-ray diffraction patterns. Here, the different colors are used to differentiate the peak intensity of the diffraction patterns. At the bottom of Figure 3, there are two nuclear peaks at the [1 1 1] and [2 0 0] positions, indexed based on the space group of *Fm-*3*m*. It is worth noting that the contribution of NiO is very weak even at *T*_A _= 400°C. The intensity of the small NiO grains (as can be seen in the SEM image) is weak and undetectable in the SR-XRD images. These characteristics (i.e., the small NiO grains) can be examined by *in situ *confocal Raman scattering, suggesting by Mironova-Ulmane *et al*. [26] of previous results obtained for NiO nanoparticles. After increasing *T*_A _we observe a significant broader peak around 2*θ *= 21.3°, which is associated with the NiO structure of the Miller index [2 0 0], and indicates the coexistence of NiO nanowalls and Ni grids. This may be explained by assuming the existence of the NiO phase, with the oxidation contribution coming from the Ni atoms in the grid. The pattern in the upper part of Figure 3 should contain, in principle, contributions mainly from the NiO phase after an increase in *T*_A _near 800°C. The X-ray diffraction patterns of the NiO nanowalls are refined using Rietveld analysis [27,28]. The preferred orientation is taken into account, as shown in Figure 4a, b, c, d, e. The diffraction pattern (black crosses) taken at various *T*_A _are shown, where the solid curve (red curve) indicates the fitted pattern. The difference (blue curve) between the observed and the fitted patterns is plotted at the bottom of Figure 4a, b, c, d, e. The obtained refined lattice parameters are shown in Table 2. Figure 4f shows the ratio of integrated intensity of the [1 1 1] and [2 0 0] of the NiO nanowalls as a function of *T*_A_. The ratio between the [1 1 1] and [2 0 0] peaks is noticeably higher than the standard value of 0.74 in NiO bulk at a lower *T*_A _and close to the standard value when *T*_A _= 800°C. This reveals that the NiO nanowalls for the Miller index [1 1 1] are oxidized more rapidly than for the other index [2 0 0]. The oxidized faces grow at a rate dependent upon the preferred crystallographic orientation of the NiO [1 1 1] faces even at micrometer thicknesses, which is in good agreement with previous observations [20]. Varghese *et al*. reported lattice fringes with an interplanar spacing of 2.44 Å corresponding to the [1 1 1] planes in the HRTEM image of the NiO nanowalls. For a face-centered cubic structure, the general order of the surface energies associated with the crystallographic planes is γ_{111} _< γ_{100} _< γ_{110}_, so the [1 1 1] facets can be easily stabilized [29]. Low surface energy could enhance the growth rates along the [1 1 1] directions, further enhancing aggregation of the NiO grains, leading to selective induction of anisotropic growth on a specific facet to form nanowalls.

**Table 2:** Summary of X-ray refinement results for NiO nanowalls

*T*_A_(°C)	Lattice parameters *a *(Å)		*χ*^2^	*R*_p _(%)	*R*_wp _(%)	Weight percentage	
	NiO	Ni				NiO (%)	Ni (%)
400	4.1832(11)	3.5305(1)	1.23	1.53	3.47	2.1(3)	98(1)
500	4.1612(9)	3.5135(2)	2.31	1.36	3.65	7.1(1)	93(2)
600	4.1821(1)	3.5295(1)	0.819	1.33	3.1	8.1(1)	92(1)
700	4.1871(3)	3.5338(1)	1.81	1.8	4.14	67(2)	33(1)
800	4.1867 (2)	3.5312 (4)	1.19	1.67	3.59	91(5)	9.1(5)

### EDS mapping

**Figure 5:**
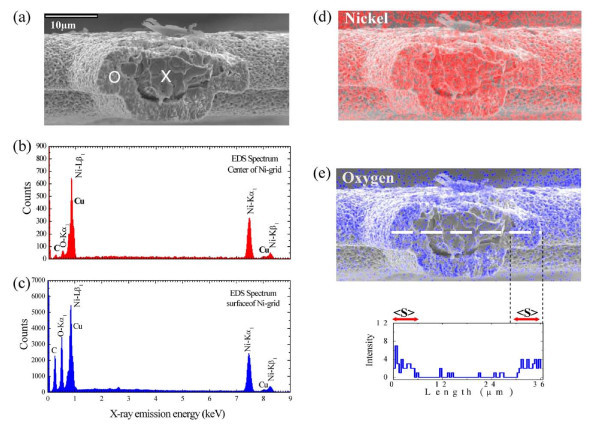
**EDS mapping of NiO**. **(a) **Side view of a cross-sectional NiO grid SEM image, indicating the existence of the Ni core and NiO surface at *T*_A _= 700°C, **(b) **Typical EDS pattern taken at the Ni core, and **(c) **NiO surface. **(d, e) **Two-dimensional EDS mapping images of the distribution of elements presented using the lock-in energy of Ni-Kα_1 _(7.3 to 7.6 keV) and O-Kα_1 _(0.4 to 0.6 keV). The inset to (e) shows a step function at both edges that can be used to define the mean diffusion length <*S *>.

Since the NiO nanowall growth process is dependent on the surface diffusion of Ni (with respect to the annealing temperature and time), the cross-section of such a grid should look like the core-shell structure of Ni/NiO. To verify the formation of NiO nanowalls, further SEM investigation was carried out. An EDS (Inca x-sight model 7557, Oxford Instruments, UK) mapping technique was used to measure the shell thickness of NiO. EDS mapping generates a two-dimensional image indicating the abundance of an element. The intensity of the image allows direct visualization of the spatial distribution of any element, such as nickel or oxygen, on the surface of the Ni grid. Figure 5a depicts an SEM image of a cross-section of the Ni grid of the selected sample (at *T*_A _= 700°C). It can be seen that the cross-section is not uniform with the formation of the core and shell being dominated by Ni and NiO, respectively. Typical EDS elemental spectra taken at the core and shell (indicated by the white cross and circle in Figure 5a are shown in Figure 5b, c, respectively. The peaks shown in Figure 5b are associated with a series of elemental Ni which can be assigned to Ni-Lβ_1_, Ni-Kα_1_, and Ni-Kβ_1 _(the oxygen peak is weak and can be ignored), verifying that the core center contains only Ni element. The small peaks of Cu and C were the result of the carbon film on the Cu grid from mounting the sample. The surface shell, because of thermal activation, showed an increase in the oxygen contribution, shown in Figure 5c. Moreover, the Ni/O ratio is estimated to be 0.91(1), which is close to the stoichiometric composition of NiO, indicating the high purity of the nanowalls and the existence of nickel vacancies. Figure 5d, e show EDS mapping images of the distribution of elements presented using the lock-in energy of Ni-Kα_1 _(7.3 to 7.6 keV) and O-Kα_1 _(0.4 to 0.6 keV), respectively. The formation of NiO nanowalls can be mapped by EDS observations and the diffusion at various points along the length of the cross-section estimated. The inset to Figure 5d shows that the length (dashed line) is dependent on the intensity of the elemental oxygen. There is an evident step function on both edges of the inset of Figure 5d. The width of the step <*s *> enables us to define the length of diffusion of the nickel at various *T*_A_. The obtained diffusion lengths are shown in Table 3. Thermal treatment of the Ni grid is known to influence the rates of oxide growth during nucleation and nanowall formation. The diffusion length is also sensitive to the thermal treatment time. A diffusion model is employed to interpret the oxidation kinetics wherein nickel transport proceeds in nickel oxide both by short-circuit and lattice diffusion.

**Table 3:** Diffusion lengths

*T*_A _(K)	<*s *> (μm)	*Q*_S_/*Q*_D_	*Q*_S _(kcal/mol)	Δ*L*_cal _(μm)
673	0.035(5)	0.427(7)	25.193	0.029
773	0.7 (2)	0.325(5)	19.175	0.689
873	1.7(2)	0.317(5)	18.703	1.616
973	6(1)	0.268(1)	15.812	5.938
1,073	9(1)	0.264(7)	15.576	9.187

NiO shell thickness along with simulated results for the lattice and short-circuit diffusion theory over the complete range of annealing temperatures.

### Short-circuit diffusion in NiO nanowalls

**Figure 6:**
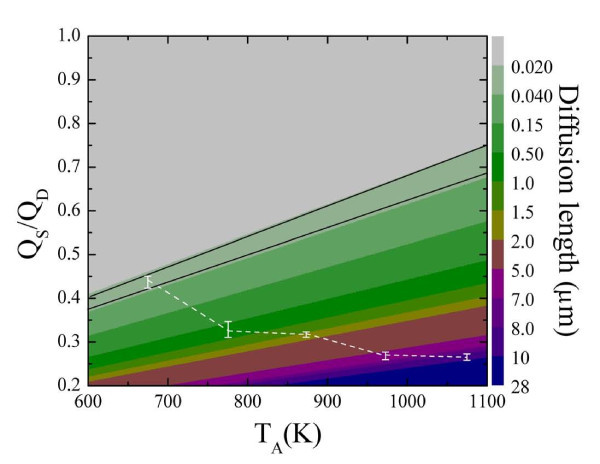
**The simulation results using *Q*_S_/*Q*_D _versus temperature**. Simulation results for the lattice diffusion theory for Ni atoms along the [1 1 1] plane, where short-circuit diffusion occurred at *Q*_S_/*Q*_D _to approximately one third. The dashed line represents the estimated length <*S *> obtained from the EDS mapping results at various *T*_A_.

The oxidation of metals in an ambient atmosphere results in the formation of a protective oxide layer on the surface of the metal, which will not allow any further diffusion of oxygen from the surface. In the case of Ni, where the diffusion coefficient of Ni is higher than that of oxygen [30], surface diffusion takes place, resulting in an increase in the NiO layer with annealing temperature. It is well-known that annealing of the Ni grid at a temperature above 500°C results in the parabolic growth of nickel oxide nanowalls. The contribution from boundary diffusion at the grain boundaries decreases with increasing nanowall size [31]. This oxidation rate is also strongly affected by line defects. This rate will be more pronounced with increased densities of line defects, where incoherent crystalline boundaries are available for short-circuit diffusion [32,33]. It is therefore important to consider the influence of the scale on Ni transport when metal facets are oxidized at temperatures less than one half the melting point of NiO. At these temperatures, recrystallization and grain growth proceed slowly; with polycrystalline oxide boundaries serving as effective short-circuit diffusion paths [34]. A wide range of growth rates may, therefore, be expected for different metal faces. The degree of short-circuit diffusion is determined by the density and crystallographic orientation of boundaries within the oriented oxide layer [35]. A simple diffusion model is employed to interpret the oxidation kinetics. In this model, Ni transport proceeds in NiO both by short-circuit diffusion at the grain boundaries and by lattice diffusion at lower annealing temperatures. The theory of lattice diffusion fully explains the diffusion mechanism [35]. The diffusion length  can be obtained from the lattice diffusivity  (square centimeter per second) according to following formula [33]:

where *β *= 8 which is the number of positions a Ni atom can jump along the [111] plane; *α *= 0.2412 nm is the *d*-spacing of the [111] plane; *D *= 2 in the denominator is the two-dimensional constant; υ*_D _*approximately 10^12 ^per second is the vibration frequency^a^; *τ *is the growth time (approximately 10,800 s); *Q*_D _= 59(1) kcal/mol is the activation energy of Ni [36]; and *R *(1.987 cal mol K^-1^) is the gas constant. The diffusion length of the NiO nanowalls is simulated based on the values of the lattice diffusion and various *T *and *Q*_S_/*Q*_D_, where *Q*_S _is the short-circuit activation energy. The simulation results using *Q*_S_/*Q*_D _versus temperature are illustrated in Figure 6. The colored bar indicates the diffusion length, and the corresponding dash line indicates the diffusion length <*S *> obtained from EDS mapping. The *Q*_S_/*Q*_D _value of approximately 0.427(7) is higher than the short-circuit diffusion predicted *Q*_S_/*Q*_D _value of approximately one third at lower *T*_A _= 400°C, revealing that the growth of NiO grains is influenced by structural changes and strain between Ni and NiO. At higher *T*_A_, corresponding to our experimental data, the value of *Q*_S_/*Q*_D _is close to 0.32(2), indicating that short-circuit diffusion is the dominant transport mechanism in the oxidation of Ni, whether oxygen or nickel is the diffusion species along short-circuit path dislocations, resulting in the growth of NiO nanowalls at the higher temperature regime. Details related to the fitting parameters are listed in Table 3.

## Phonon and magnon study of NiO nanowalls

### Superexchange of Ni-O-Ni

In the study of the system of NiO nanowalls, it is of interest to directly observe the influence of the nanowall size on the coupling strength from the phonon vibration and magnon excitation. Confocal Raman spectroscopy has the high spatial resolution and sensitivity necessary for probing the local atomic vibration and multimagnon interaction below the Neel temperature. The multimagnon properties of NiO nanowalls were investigated using a confocal Raman spectrometer (Alpha 300, WiTec Pte. Ltd., Germany) equipped with a piezo scanner and 9100 microscope objectives (n.a. = 0.90; Nikon Imaging Japan Inc., Japan). The samples were excited with a 488-nm Ar ion laser (CVI Melles Griot, Carlsbad, USA) (5 mW laser power), to form a spot 0.3 μm in diameter, giving a power density of 100 mW cm^-2^. Here configurations of two and higher order magnon excitation in NiO nanowalls are mainly determined by the dominating superexchange interaction in the Ni^2+^-O^2-^-Ni^2+ ^linear atom chains and nano-sized effects. The incident photon is virtually absorbed in an electric dipole transition process that results in the magnon excitation. The exchange mechanism through the oxygen *p*-orbital then produces a spin flip in the excited state, and a second photon is virtually emitted leaving the system with a magnon excitation. The resultant momentum transfer can exceed 1 cm^-1 ^for second-order Raman scattering. The two-magnon excitation is mainly dominated by the NNN Ni ions along the [1 0 0] direction, and is presented in terms of the antiferromagnetic exchange energy *J*_2 _(approximately 221 K), which is much stronger than the nearest-neighboring ion exchange energy  (approximately 15.7 K) in the same [1 1 1] plane of ferromagnetic coupling. Furthermore the  (approximately 16.1 K) between the nearest neighbor in adjacent [1 1 1] planes (normally antiferromagnetically aligned) which can thus be ignored and canceled out in an ideal structure [4]. The experimental values of *J*_2 _as presented in previous reports by Dietz's group [8,37] (Raman scattering) below *T*_N _are 18.5 meV in bulk NiO. Multimagnon Raman scattering in NiO is well described by the spin-wave theory utilizing the Green function formalism for an *S *= 1 antiferromagnet. The frequency of the two-magnon Raman line is estimated to be proportional to the superexchange integral *J*_2_. We measured the annealing temperature dependence of the two- and four-magnon Raman frequencies. This allows us to find out the magnetic exchange coupling and determine the possible configuration of short range four-magnon models.

### Confocal Raman scattering of NiO nanowalls

**Figure 7:**
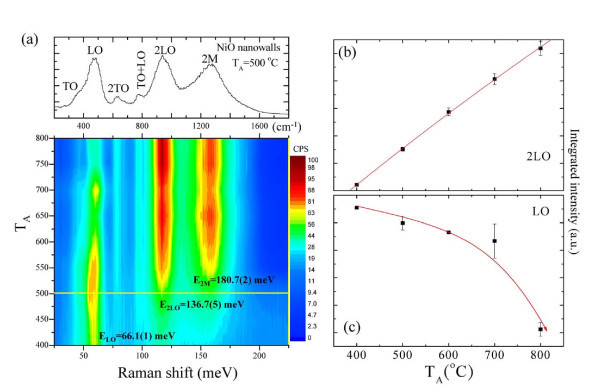
**The growth temperature *T*_A _dependence of Raman patterns**. **(a) **Two-dimensional map of the intensity and *T*_A _dependence of Raman patterns taken at room temperature. A selected Raman pattern taken at *T*_A _= 500°C is shown at the top, revealing a series of phonon modes and magnon excitations. **(b) **The growth temperature *T*_A _dependence of the integrated intensity in the 2LO and **(c) **LO mode.

Figure 7a shows the series of Raman spectra (in the bottom of the milli-electron-volt unit and top of the per-centimeter unit) taken at room temperature for annealing temperatures ranging from 400 to 800°C. Two typical one-phonon (TO and LO modes) and two-phonon (2TO, TO + LO, and 2LO modes) excitations were observed at *T*_A _= 500°C (shown at the top of Figure 7a), and are in good agreement with the values reported for bulk NiO single crystals [37]. The growth temperature *T*_A _dependence of the phonon and two-magnon peak positions and intensities obtained from two-dimensional Raman images of NiO nanowalls are shown at the bottom of Figure 7a. Here, different colors are used to differentiate the peak intensity of the Raman patterns after *T*_A _= 400°C to 800°C. As indicated on the bottom of Figure 7a, there is one-phonon peak at *E*_LO _= 66.1(1) meV, corresponding to the LO mode and decreasing with increasing *T*_A_. After *T*_A _is increased to 500°C, we observed two significant broader peaks around *E*_2LO _= 136.7(5) and 180.7(2) meV, respectively, which are associated with the 2LO and two-magnon modes and increase as *T*_A _increases. The anomalous behaviors can be analyzed quantitatively using the profile fitting method. These peaks, including the phonon and two-magnon modes, were analyzed by the Voigt function covering the whole regime. The detailed *T*_A _dependences of the peak position and full widths at half maximum (FWHM) are listed in Table 4. Figure 7b shows the *T*_A _dependence of the integrated intensity of the selected peak for the 2LO mode. As the growth temperature *T*_A _is reduced, the integrated intensity of the 2LO mode rapidly decreases at around *T*_A _= 400°C, signaling the finite size effect, which acts to confine the lattice vibration in corroboration with the strength of two-phonon coupling. Figure 7c shows that the integrated intensity increases in the single phonon LO mode with decreasing *T*_A_, in comparison with that in two-phonon 2LO mode. The enhancement of intensity that occurs at lower growth temperatures is due to parity-breaking defects, since the concentration of nickel vacancies is high, as can be quantitatively investigated through two-magnon Raman scattering.

**Table 4:** Summary of the Voigt fitting parameters for one- and two-phonon mode of NiO nanowalls

*T*_A_(°C)	TO (meV)		LO (meV)		2TO (meV)		TO + LO (meV)		2LO (meV)	
	Center	FWHM	Center	FWHM	Center	FWHM	Center	FWHM	Center	FWHM
400	56.3	27.6	66.1	13.5	90.4	22.2	113.0	19.8	135.8	24.3
500	50.0	12.0	66.3	15.4	91.5	16.6	112.8	7.8	136.7	22.6
600	52.9	15.3	68.5	13.4	91.9	11.1	112.6	5.2	137.6	21.7
700	53.2	16.0	69.5	12.9	91.6	12.1	112.8	5.8	137.6	21.7
800	50.3	9.4	67.5	15.0	92.0	11.0	112.8	5.2	137.4	21.8

### Magnon excitation of NiO nanowalls

**Figure 8:**
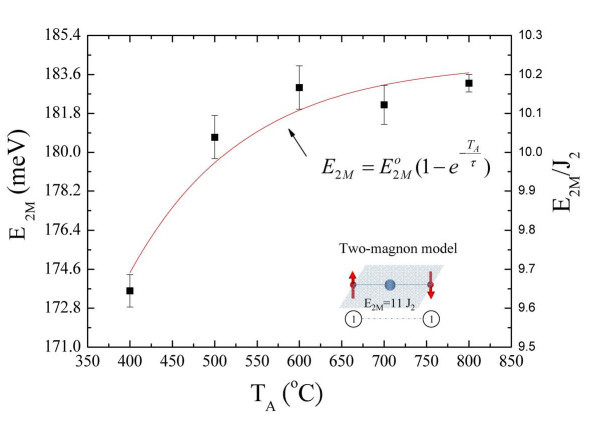
**Growth temperature *T*_A _dependence of the *E*_2 M _peak**. The inset shows a possible model for the two-magnon configuration and minimum energy as predicted by an Ising cluster model calculation, where the linkages denote the exchange interactions and numbers give the number of spin deviations on each site.

It can be inferred that the additional peak visible just below the phonon modes (labeled as 2 M) is magnetic in origin, since it vanishes above the Neel temperature [15]. The extrapolation of the *T*_A _dependence of the intensity of the two-magnon frequency to *I*_2 M _to approximately 0 gives the growth temperature *T*_A _approximately 400°C. This signals the start of a spin correlation which is much weaker than that of the Bulk NiO. The growth temperature *T*_A _dependence of the *E*_2 M _peak is shown in Figure 8. The solid curve describes an exponential growth function for the magnetic short-range correlation, namely , where  and *τ *= 131(4)°C represent the final energy and fitted parameters, respectively. Furthermore, the deviation of the two-magnon energy with growth temperature, defined by *G *= d*E*_2*M*_/d*T*_A_, can be used to probe the two-magnon correlation growth rate. Thus, when *T*_A _= 400°C to 600°C, the peak shift rate would be 1.4 meV/°C. The increase in energy of the two-magnon growth rate with increasing *T*_A _can be explained by the increase in the spin correlation length, while the smaller intensity of the Raman response caused by the Ni^2+^-O^2-^-Ni^2+ ^superexchange mechanism is associated with decreased amounts of NiO nanowalls due to the finite size effect. The reduced coordination of surface spin and the incomplete compensation between the antiferromagnetic sublattices will also cause a fundamental decrease in the magnetic ordering in the NiO nanowalls. These characteristics (i.e., the lower intensity) agree with results previously reported for NiO nanoparticles by Mironova-Ulmane *et al*. [26]. In their comprehensive analysis, they found that the intensity of the two-magnon peak decreases with particle size, but would, upon heating subsequently shift to lower energies and broaden. Furthermore, it is well-known that the lattice strain will introduce a slight rhombohedral distortion. The small distortion will create a difference in the anisotropic energy gap between  and  which is associated with the rhombohedral contraction occurring at lower *T*_A_. This will contribute to line broadening at the magnon peak. The inset to Figure 8 shows a possible model for the two-magnon configuration and minimum energy as predicted by the Ising cluster model calculation, where the linkages denote the exchange interactions and numbers give the number of spin deviations on each site. Chinn *et al*. reported the two-magnon models, the simple cubic lattice, for bulk KNiF_3 _[38]. There are three transformations of the , , and  states from two-magnon excitations, but only the  states have a nonzero spectral density for a scattering element matrix of the form , where the sum includes the next-nearest-neighbors in the case of NiO. Utilizing Chinn's  Green's function, Dietz *et al*. [12] reported a value of *J*_2 _approximately 18 meV for bulk NiO. The strong two-magnon peak at *E*_2 M _= 183.2(2) meV with <*d *> = 416(18) nm is due to the two-magnon excitation, which is nearly ten times that of *J*_2_. This is in agreement with the values previously reported for bulk NiO [8,37], but smaller than the predicted value of 11 from the Ising cluster model. The mean number of *N*_2 M _obtained from *N*_2 M _*= E*_2 M_/*J*_2 _with the next-nearest-neighbor exchange *J*_2 _= 18 meV, shown in Table 5 may be associated with the concentration of nickel vacancies. According to previous Ni_c_O doping nonmagnetic ion models [39], the two-magnon peak can be roughly expressed as , where *c *is the chemical composition of nickel and *x *= (1-*c*)% is defined as the concentration of nickel vacancies; *z *= 6 is the number of next-nearest-neighbor; *J*_2 _= 18 meV is the superexchange interaction energy within 180° of the Ni^2+^-O^2-^-Ni^2+ ^atomic chain; and *S *= 1 is the antiferromagnetic spin. The values obtained for *x *for the Ni_c_O nanowalls are listed in Table 5. The results are consistent with the Ni_1-*x*_Mg*_x_*O system [40,41]. Lowering the local symmetry at the Ni^2+ ^sites caused by the chemical substitution and vacancies will result in shifting of the two-magnon peaks. It is worth noting that the one-magnon Raman frequency has a very weak reported value of 38 cm^-1^, which is undetectable in the study of NiO nanowalls [40].

**Table 5:** Summary of the Voigt fitting parameters for two- and four-magnon mode of NiO nanowalls

*T*_A _(°C)	2 M (meV)		4 M (meV)		*x *(%)	*R *= *E*_4 M_/*E*_2 M_
	Peak	FWHM	Peak	FWHM		
400	173.6	51.8	316.0	131.1	13.6	1.818
500	180.7	37.6	320.5	83.5	9.6	1.773
600	183.0	35.0	322.8	62.7	8.3	1.763
700	182.2	36.7	323.1	62.4	8.8	1.773
800	183.2	33.8	323.6	60.0	8.3	1.766

**Figure 9:**
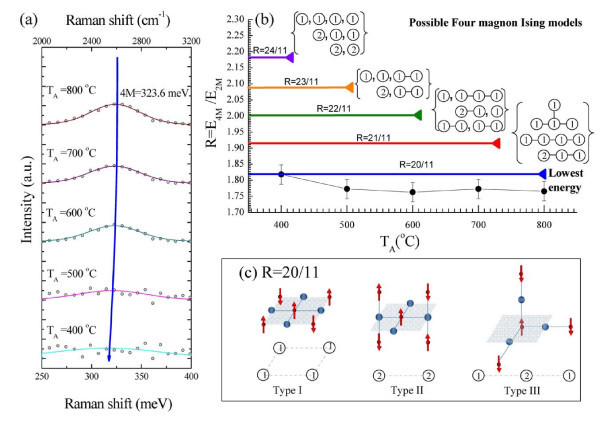
**Mechanism of four-magnon excitation**. **(a) **Growth temperature *T*_A _dependence of the *E*_4 M _peak, **(b) **evolution of the scattering factor *E*_4 M_/*E*_2 M _at various growth temperatures *T*_A_. There is no obvious change observed in the growth temperature dependence *T*_A _of *R *and a lowest state is obtained around *R *= 20/11 in this study. **(c) **Schematic plot of three possible four-spin deviations in the Ising states at sites connected by strong superexchange interactions in a simple cubic lattice. The circles indicate sites; the number gives the number of spin deviations at each site, while the linkages denote an exchange interaction between next-nearest-neighbor sites.

The weak and broad peak at 323.6 meV is assigned to the four-magnon excitation of NiO nanowalls (marked as 4 M) and presented in Figure 9a. As *T*_A _is decreased from 800°C to 400°C, the 4 M peak shifts to a lower energy and broaden as a results of the nanosized effect. Moreover, the ratio of the growth temperature *T*_A _dependence of the four-magnon states to the two-magnon states gives a scattering factor value of *R *= *E*_4 M_/*E*_2 M_, as shown in Figure 9b and Table 5. This can be used as an indicator of the four-magnon configuration from the lowest to higher states. In general, the Ising calculation is used for prediction in a lot of four-magnon models, such as the lowest state of 20*J*_2 _(*D *= 3) and four higher states of 21*J*_2 _(*D *= 3), 22*J*_2 _(*D *= 3), 23*J*_2 _(*D *= 2), and 24*J*_2 _(*D *= 3), where *D *is the degeneracy. The related theoretical scattering factors *R *with corresponding four-magnon configurations are plotted in to Figure 9b, where the lowest two-magnon energy used in the calculation is 11*J*_2_. There is no obvious change observed in the growth temperature dependence *T*_A _of *R *and a lowest state is obtained around *R *= 20/11 in this study. Three out of four possible magnon models at lowest state, proposed in principle by the Ising model plus second-order perturbation theory [37], are shown in Figure 9c. A schematic plot of the three possible four-spin deviation Ising states on sites were connected by strong superexchange interactions in a simple cubic lattice. The circles indicate sites; the number gives the number of spin deviations on each site. The linkages denote an exchange interaction where the sites are next-nearest-neighbor. The differences between the experimental and theoretical results can be explained by neglecting the weak ferromagnetic coupling and constant *J*_2 _for all compositions in the system. The finite size effect and nickel vacancy dependence of the Raman phonon-magnon modes most likely play the dominant role affecting the Raman shifts that lead to unusual properties in NiO nanowalls.

## Conclusion

The chemical vapor deposition technique was successfully utilized to grow NiO nanowalls on a Ni grid with various mean widths without using any catalyst. The growth temperature for the NiO nanowalls was confined from 400°C to 800°C, which is 0.275 and 0.55 times the Ni melting point, following the parabolic rate law of Wagner's scaling theory. X-ray refinement reveals that the NiO nanowalls with the Miller index [1 1 1] oxidized more rapidly than with the other index [2 0 0], and the oxidized faces grow at a rate dependent upon the crystallographic preferred orientation of the NiO [1 1 1] faces. The length of diffusion of nickel along the [1 1 1] plane at various growth temperatures can be obtained from EDS mapping. The results agreed with the short-circuit diffusion mechanism simulation. Confocal Raman scattering was utilized to study the phonon and magnon configurations for these samples. The appearance of integrated intensity for the one- and two-phonon modes reflects the existence of the finite size effect and nickel vacancies. Two- and four-magnon excitations generated in NiO nanowalls may help to identify the Ni^2+^-O^2-^-Ni^2+ ^superexchange mechanism associated with the short-range magnetic interactions and magnon configurations.

## Competing interests

The authors declare that they have no competing interests.

## Authors' contributions

SYW wrote, conceived, and designed the experiments. ACG grew the samples and participated in the phonon and magnetic characterization. C-YH and CCY analyzed the data. TSC, C-LC, and Y-RM contributed the experimental facility of SR-XRD, Raman, and FE-SEM, respectively. All authors discussed the results, contributed to the manuscript text, commented on the manuscript, and approved its final version.

## Endnotes

^a^The vibrational frequency of Ni atom is defined as follows: , where α = 0.2412 nm is the *d*-spacing of the [1 1 1] plane, *m *= 74.7 g/mol is the Ni molar weight, and *Q*_D _= 59(1) kcal/mol is the activation energy of Ni.
